# Comparative transcriptome profiling of virulent and avirulent isolates of *Neoparamoeba perurans*

**DOI:** 10.1038/s41598-022-09806-5

**Published:** 2022-04-07

**Authors:** Aaron J. Smith, Philip B. B. Crosbie, Barbara F. Nowak, Andrew R. Bridle

**Affiliations:** Institute for Marine and Antarctic Studies, University of Tasmania, Launceston, TAS Australia

**Keywords:** Transcriptomics, Parasitology

## Abstract

*Neoparamoeba perurans*, the aetiological agent of amoebic gill disease, remains a persistent threat to Atlantic salmon mariculture operations worldwide. Innovation in methods of AGD control is required yet constrained by a limited understanding of the mechanisms of amoebic gill disease pathogenesis. In the current study, a comparative transcriptome analysis of two *N. perurans* isolates of contrasting virulence phenotypes is presented using gill-associated, virulent (wild type) isolates, and in vitro cultured, avirulent (clonal) isolates. Differential gene expression analysis identified a total of 21,198 differentially expressed genes between the wild type and clonal isolates, with 5674 of these genes upregulated in wild type *N. perurans*. Gene set enrichment analysis predicted gene sets enriched in the wild type isolates including, although not limited to, cortical actin cytoskeleton, pseudopodia, phagocytosis, macropinocytic cup, and fatty acid beta-oxidation. Combined, the results from these analyses suggest that upregulated gene expression associated with lipid metabolism, oxidative stress response, protease activity, and cytoskeleton reorganisation is linked to pathogenicity in wild type *N. perurans*. These findings provide a foundation for future AGD research and the development of novel therapeutic and prophylactic AGD control measures for commercial aquaculture.

## Introduction

*Neoparamoeba perurans* is a free-living, facultative parasite of a variety of marine farmed aquaculture species^1^, and the aetiological agent of amoebic gill disease (AGD)^2^. A potentially lethal gill disorder without therapeutic intervention^3,4^, progression of AGD compromises normal gill function, as *N. perurans* interactions with gill epithelia result in hyperplastic mucoid patches and subsequent loss of respiratory and osmoregulatory structure^5^. Although amoebae from the genus *Neoparamoeba* appear to be ubiquitously distributed in the coastal marine environment^6^, prevalent outbreaks of AGD were initially limited to Tasmanian Atlantic salmon mariculture operations^3^. However, throughout the last decade, AGD has emerged globally as a significant source of economic loss and a threat to animal welfare in commercial Atlantic salmon aquaculture, with outbreaks now reported in most Northern and Southern Hemisphere salmon producing regions^1,7^.

Commercially viable therapeutic control measures for AGD in contemporary Atlantic salmon aquaculture are limited to non-medicinal freshwater or hydrogen peroxide bathing treatments^8,9^, typically administered via well-boat. While both treatments are effective in reducing AGD-associated pathology in affected cohorts, a subset of gill-associated *N. perurans* typically survive exposure, facilitating rapid post-bathing proliferation and reinfection of the host^8,10,11^. To mitigate AGD progression in sea farmed Atlantic salmon, 8-to-13- bathing treatments are required over the duration of a 15-to-18-month marine production cycle^12,13^. Current estimates of the operational costs imposed by labour and infrastructure requirements, and cumulative handling mortalities inherent to reiterative bathing treatments over a production cycle^13^, are not publicly available. However, early estimates placed the economic burden of AGD, including associated loses and freshwater bathing treatments, at 10–20% of total production costs^14^. Given the high associated costs, and, in the case of freshwater treatments, heavy reliance on finite freshwater supplies, current bathing treatments are not considered long-term solutions for the industry^13^.

Development of alternative therapeutic AGD mitigation strategies has been met with limited experimental success, with few achieving results comparable to freshwater or hydrogen peroxide treatments^15–17^. Despite decades of research, oral delivery of the anthelminthic, bithionol, or L-cystine ethyl ester have been the only alternatives to therapeutic bathing methods to achieve comparable protective efficacy against *N. perurans*^17,18^. While vaccination is considered key to effective disease control in intensive finfish culture^19,20^, owing to their biological complexity and the intricacies of host-parasite interactions, identification of protective antigens from fish parasites has remained elusive^20^. Accordingly, to date, administration of experimental vaccine candidates to Atlantic salmon has elicited, at best, a marginal protective antibody response against *N. perurans*^21–26^. Until recent advances, the limited progress made towards identifying the mechanisms by which *N. perurans* induce AGD has actively hindered development of alternative methods of AGD control.

In the absence of a publicly available reference genome, de novo transcriptome assembly and proteomic profiling provide valuable tools for the identification of *N. perurans* virulence determinants. Proteomic analyses have revealed distinct protein expression profiles between virulence-attenuated and malt yeast agar (MYA) cultured wild type *N. perurans* isolates^27,28^. With focus on soluble, cytoplasmic content, upregulated expression of proteins supporting wild type *N. perurans* cytoskeletal re-organisation, protein synthesis, response to oxidative stress, and putative host immunomodulation have been reported^27^. Exoproteomic analysis of cultured wild type *N. perurans* demonstrated serine protease activity in the extracellular product of virulence-attenuated *N. perurans*, in which the *N. perurans* commensal microbiome was partially attributed. Notably, in contrast to a previous in vitro observation^29^, this cell cytopathic effect was found to be absent in virulent *N. perurans* cultures^28^. Combined transcriptomic profiling of Atlantic salmon and cultured wild type *N. perurans* has been conducted, wherein a theoretical model for the host-parasite interactions involved in AGD pathogenesis was described^30^. However, transcriptomic analysis comparing *N. perurans* isolates with clear differences in their degree of virulence and comparisons using gill harvested wild type *N. perurans* not subject to serial passage has yet to be conducted.

Here, a comparative transcriptomic analysis of virulent wild type and avirulent clonal *N. perurans* isolates is presented. In contrast to wild type *N. perurans*, harvested directly from the gills of AGD-infected Atlantic salmon, the clonal strain of *N. perurans* used in the current study has been observed to be non-pathogenic towards Atlantic salmon *in vivo*^29^. This process of virulence attenuation in *N. perurans* through serial passage in vitro has been replicated by other research groups^27,31^. Common to these virulence-attenuated isolates is the absence of AGD-related gill pathology and gill-associated *N. perurans* detection in challenged Atlantic salmon^27,29,31^, despite detection of trophozoites in the water column of challenge systems^29,31^. Leveraging observed differences in pathogenicity, the current study aimed to characterise gene expression changes between wild type and clonal *N. perurans* to elucidate potential determinants of virulence, and to identify putative therapeutic or vaccine candidates for future characterisation and validation.

## Results

### Transcriptome assembly quality

Six *N. perurans* samples (three wild type and three clonal isolates) totalling 382 million pairs of 101 base length paired-end reads were generated from an Illumina Hiseq sequencing platform. Following pre-processing of samples prior to transcriptome assembly, a total of 378 million pairs from these six *N. perurans* samples were retained for assembly of contigs (Table S1B and C). From the Trinity assembly, a total of 86,517 transcripts clustered into 66,361 putative gene groupings (hereinafter referred to as genes for brevity) were generated (median transcript length: 507 bases, N50: 1155 bases; Table S1A). Evaluation based on BUSCO found 216 (84.7%) core, eukaryotic ortholog genes represented in the *N. perurans* transcriptome, with 27.8% duplicated contigs and a small percentage of fragmented or missing genes (C:84.7% [S:56.9%, D:27.8%], F:8.2%, M:7.1%, n:255). The high number of core eukaryotic ortholog genes is indicative of the relative completeness of the *N. perurans* transcriptome presented in the current study.

### Transcriptome annotation

Transcripts were annotated using Trinotate leveraging Blastx to homology search the Swiss-Prot and the NCBI non-redundant (NR) databases, resulting in 28,840 (33.3%) and 41,550 (48.0%) positive identities, respectively. Approximately 14.1% of annotated transcripts were assigned best hit to sequences of the top five eukaryotic species, including *Perkinsela* sp. CCAP 1560/4, *Acanthamoeba castellanii* str. Neff, *Arabidopsis thaliana*, *Dictyostelium discoideum* and *Symbiodinium microadriaticum*. Of the transcripts assigned best hit using the NCBI NR database, approximately 35.1% were assigned to uninformative hypothetical, or uncharacterised proteins. Utilising Blastx and Blastp homology searches against the Swiss-Prot database and pfam2go, a total of 28,238 (98.0%) annotated transcripts were assigned to at least one GO term belonging to the three GO domains: biological process (63.9%), molecular function (22.2%), and cellular component (14.9%).

### Differential gene expression analysis

**Figure 1:**
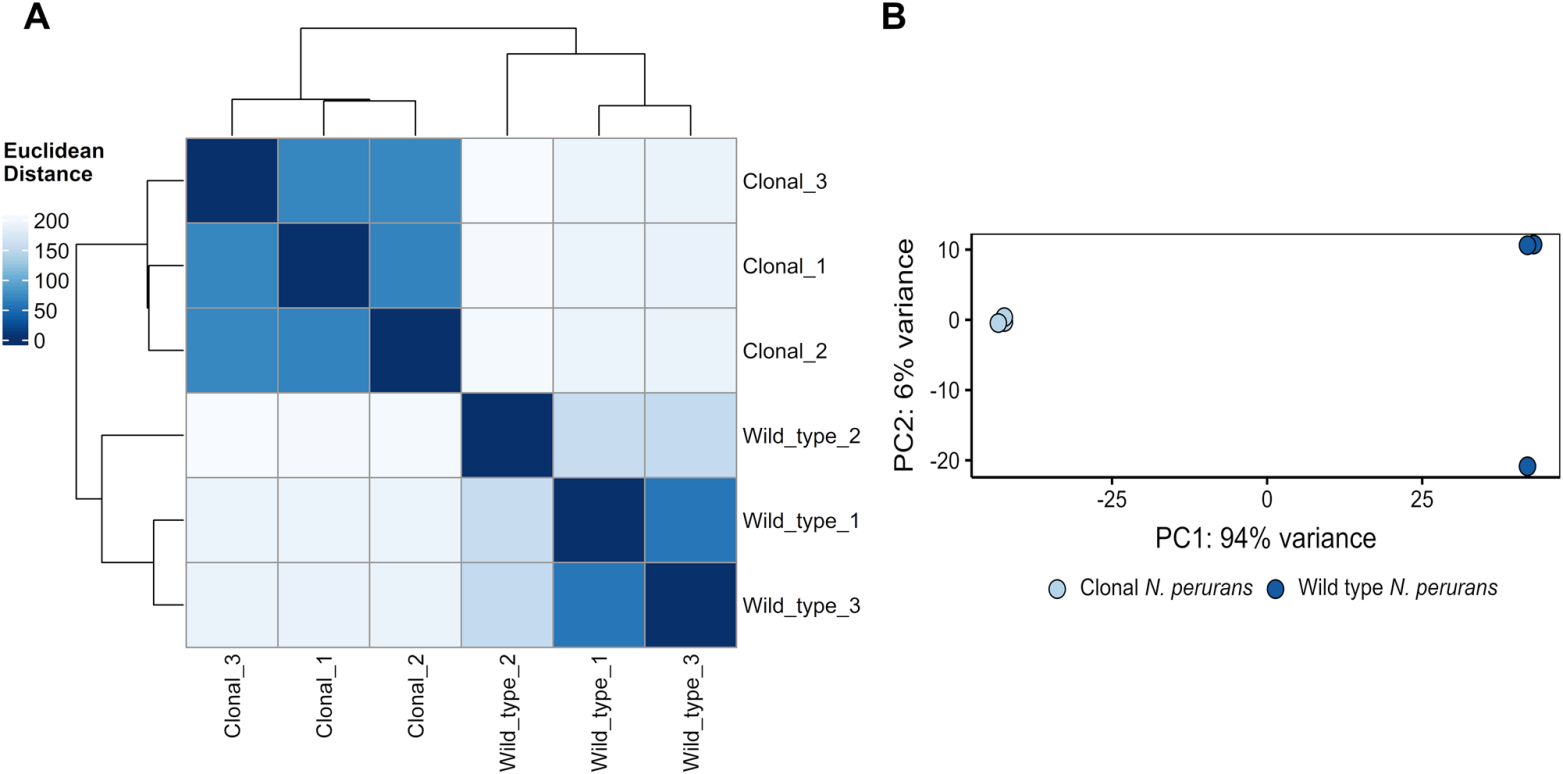
Heatmap (**A**) of sample-to-sample differences and Principal Component Analysis (PCA) (**B**) of the top 500 most variable genes using normalised (variance stabilising transformation) RNAseq data generated following raw read processing and de novo assembly using Trinity. Jitter was added to the PCA plot to avoid overplotting of replicates.

**Figure 2:**
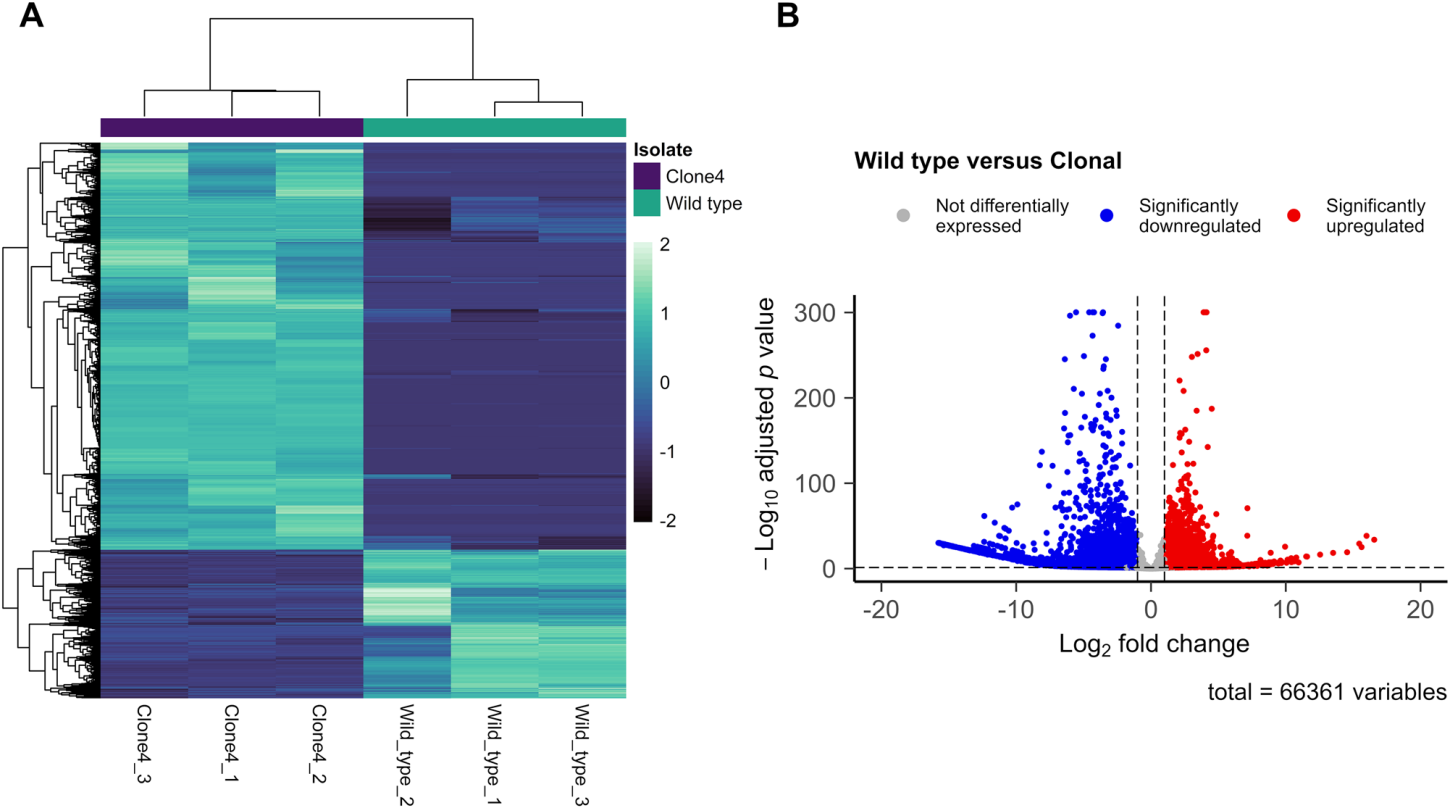
Heatmap (**A**) and volcano plot (**B**) of all differentially expressed genes comparing wild type versus clonal *N. perurans* isolates. Heatmap shading delineates the difference in Z-score between differentially expressed genes. A full list of differentially expressed genes is included in Table S2.

Principal component analysis and clustering of normalised gene counts revealed distinct variation between the expression profiles of wild type and clonal *N. perurans* samples and expected clustering between biological triplicates, with some deviation observed between wild type replicates (Fig. 1). No significant outliers were detected, and all RNAseq samples were retained for downstream analysis. On average, of the 66,361 genes predicted, 41,583 (62.7%) were expressed in the wild type isolates, while 57,937 (87.3%) were expressed in the clonal isolates. Facilitating a comparative approach to detect transcripts potentially associated with wild type *N. perurans* pathogenicity, differential gene expression analysis identified 21,198 genes differentially expressed between the wild type and clonal isolates (Wald test; Benjamini–Hochberg adjusted *P* value < 0.05 and log2 fold change > 2). Of these genes, 5,674 were found to be upregulated in wild type *N. perurans*, with the remaining 15,525 genes showing downregulated expression (Fig. 2; Table S2). Annotation of differentially expressed genes in wild type *N. perurans,* leveraging the Swiss-Prot database, resulted in a total of 7,921 (37%) positive identities. When leveraging the NCBI Nr database, the percentage of annotated gene increased to 53%, with a total of 11,232 positive identities.

**Figure 3:**
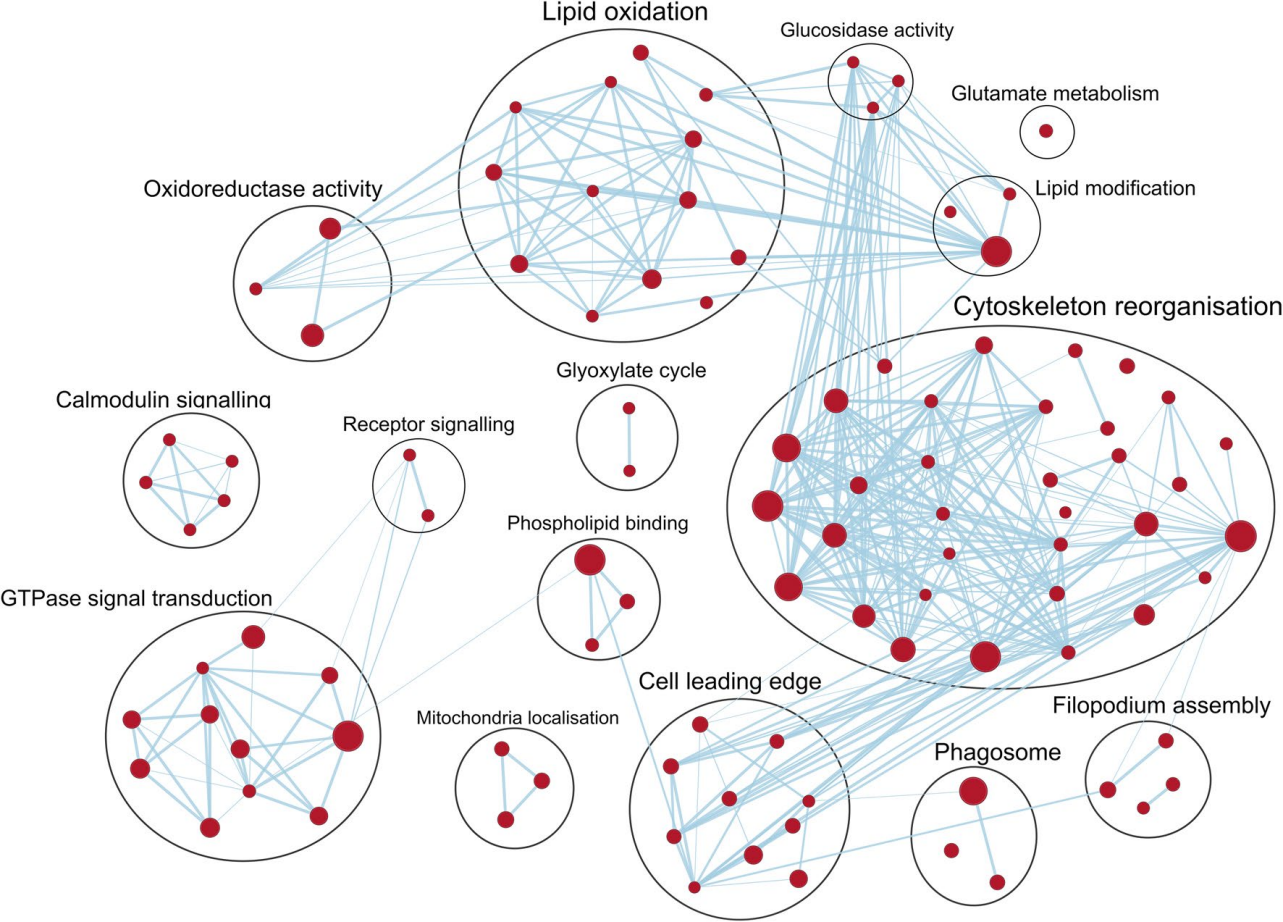
Gene set enrichment analysis delineating the gene sets (nodes) significantly enriched wild type N. perurans isolates with respect to the biological process, molecular function, and cellular component GO domains. Node size reflects the total number of genes within the gene set. Edge thickness of lines between nodes represents the degree of similarity between gene sets (Overlap Coefficient = 0.5). A full list of genes associated with the upregulated gene sets of the wild type isolates is included in Table S4.

Upregulated differentially expressed genes, along with genes associated with enriched pathways (presented in Sect. "Gene set enrichment analysis"; Fig. 3) in wild type *N. perurans* isolates, were assigned to four categories of potential, putative virulence determinants inferred from other parasitic amoeba species. These broadly defined categories included genes associated with *N. perurans* cytoskeleton reorganisation, response to oxidative stress, protease activity, and lipid metabolism. Upregulated genes encoding for putative virulence factors falling under these broadly defined categories are summarised in Table 1.

**Table 1:** A non-exhaustive list of putative virulence factors upregulated in wild type *N. perurans* identified through comparative transcriptome analysis of virulent and avirulent *N. perurans* isolates. A complete list of differentially expressed genes, including gene name or identifier, is located in Table S2.

Classification	Putative trinity gene	BLAST identification	Scientific name	Log2FC	% identity	E-value
Cytoskeleton reorganisation	TRINITY_DN928_c0_g1	Actin, plasmodial isoform	*Physarum polycephalum*	3.5	96.5	3E-265
	TRINITY_DN8982_c0_g1	Beta actin, partial	*Paramoeba pemaquidensis*	2.9	100	1E-33
	TRINITY_DN38356_c0_g1	Severin	*Cavenderia fasciculata*	4.9	56.9	2E-131
	TRINITY_DN1132_c0_g1	Gelsolin repeat-containing protein	*Acanthamoeba castellanii* str. Neff	1.6	28.0	6E-42
	TRINITY_DN16647_c0_g1	Villin	*Heterostelium album* PN500	2.1	38.8	7E-30
	TRINITY_DN6068_c0_g2	Myosin1, putative	*Acanthamoeba castellanii* str. Neff	1.7	49.6	0E + 00
	TRINITY_DN47507_c0_g1	Actin binding protein	*Tieghemostelium lacteum*	4.1	43	8E-26
	TRINITY_DN553_c0_g1	Profilin-A	*Physarum polycephalum*	2.9	57.1	7E-42
	TRINITY_DN26363_c0_g1	Gelation factor, putative	*Acanthamoeba castellanii* str. Neff	3.9	41.5	1E-141
	TRINITY_DN33745_c0_g1	Formin domain containing protein	*Acanthamoeba castellanii* str. Neff	2.1	27.8	6E-26
	TRINITY_DN2239_c0_g1	Cofilin/tropomyosin-type actin-binding protein	*Geosmithia morbida*	3.5	39.4	4E-16
Elongation Factors	TRINITY_DN144_c0_g3	Translation elongation factor 1-alpha	*Colpoda inflata*	9.1	87.3	0E + 00
	TRINITY_DN11111_c0_g1	Elongation factor 2, putative	*Tetrahymena thermophila* SB210	7.9	71.4	0E + 00
	TRINITY_DN32109_c0_g1	Elongation factor Tu	*Novosphingobium aerophilum*	2.3	66.5	0E + 00
	TRINITY_DN10393_c0_g1	Translation elongation factor Ts	*Rhodospirillaceae bacterium*	1.9	31.1	2E-30
Protease activity	TRINITY_DN24105_c0_g1	Peptidase S8 and S53 domain-containing protein	*Neoparamoeba perurans*	2.5	87.5	0E + 00
	TRINITY_DN7604_c5_g1	Physaropepsin	*Heterostelium album* PN500	2.3	41.8	4E-138
	TRINITY_DN28894_c0_g1	Cathepsin B	*Naegleria fowleri*	1.0	38.6	2E-38
	TRINITY_DN25447_c0_g1	Aspartic proteinase-like protein 2 isoform 2	*Planoprotostelium fungivorum*	1.6	38.8	3E-65
	TRINITY_DN38736_c0_g1	Cathepsin Z	*Salpingoeca rosetta*	1.3	51.2	4E-102
Response to oxidative stress	TRINITY_DN13252_c0_g1	Cu–Zn superoxide dismutase	*Apostichopus japonicus*	2.0	67.6	1E-51
	TRINITY_DN3519_c0_g1	Manganese and iron superoxide dismutase	*Acanthamoeba castellanii* str. Neff	1.5	52.5	4E-66
	TRINITY_DN31099_c0_g1	Copper/zinc superoxide dismutase	*Acanthamoeba castellanii* str. Neff	1.2	30.7	4E-27
	TRINITY_DN26761_c0_g1	Cysteine peroxiredoxin	*Lichtheimia corymbifera JMRC:FSU:9682*	3.9	53.02	2E-81
	TRINITY_DN5100_c0_g1	Peroxiredoxin-2	*Peromyscus maniculatus bairdii*	2.8	63.9	9E-83
	TRINITY_DN7459_c0_g2	Glutathione peroxidase	*Chattonella marina* var. *antiqua*	3.7	50.6	7E-12
	TRINITY_DN37673_c0_g1	Heme peroxidase	*Coccomyxa subellipsoidea* C-169	4.7	62.2	1E-121
Lipid metabolism	TRINITY_DN3675_c1_g1	Phosphlipidase D2	*Craterostigma plantagineum*	3.0	48.2	1E-128
	TRINITY_DN43561_c0_g1	Phospholipase D1	*Craterostigma plantagineum*	2.2	48.4	3E-127
	TRINITY_DN29373_c0_g1	POX5 (Acyl-coenzyme A oxidase 5)	*Symbiodinium microadriaticum*	3.8	55.9	5E-178
	TRINITY_DN24593_c0_g1	Hydroxyacyl-coenzyme A dehydrogenase	*Pythium insidiosum*	2.6	56.08	1E-129
	TRINITY_DN8529_c0_g1	SCP2 sterol transfer family protein	*Acanthamoeba castellanii* str. Neff	2.3	48.9	4E-50
	TRINITY_DN17074_c0_g1	3-hydroxybutyryl- dehydratase	*Nannochloropsis gaditana*	2.2	51.8	5E-72
	TRINITY_DN8529_c0_g1	SCP2 sterol transfer family protein	*Acanthamoeba castellanii* str. Neff	2.3	48.9	4E-50
	TRINITY_DN47585_c0_g1	Thiamine pyrophosphate-binding protein	*Deltaproteobacteria bacterium*	2.4	71.3	7E-62

Comparison = wild type versus clonal; FC = fold change; E-value = expect value.

### Gene set enrichment analysis

Using gene expression data from the entire *N. perurans* transcriptome, GSEA querying gene sets from the three GO domains, biological processes, molecular function, and cellular component, revealed 978 / 5354 gene sets putatively assigned by homology were upregulated in wild type *N. perurans* isolates. Of these gene sets, 206 were significantly enriched at a nominal *P* value of < 0.001 (Table S3). Gene sets potentially linked with putative virulence factors in wild type *N. perurans* isolates were identified using GSEA. Highly enriched gene sets putatively associated with actin-cytoskeleton in *N. perurans*, included cortical actin cytoskeleton (cellular component) and actin-binding, with associated gene sets therein, including actin filament binding (molecular function), actin crosslink formation, and actin filament bundle assembly (biological process). Additional highly enriched gene sets associated with mechanisms putatively mediated by actin-myosin processes in *N. perurans* were also significantly enriched, including pseudopodia, macropinocytic cup (cellular component), and phagocytosis (biological process). Finally, processes putatively associated with lipid metabolism in *N. perurans* were highly enriched in wild type isolates, involving gene sets for fatty acid beta-oxidation and the associated child terms, fatty acid beta-oxidation acyl-CoA oxidase, (biological process), and acyl-CoA oxidase activity (molecular function). Genes included in the enriched gene sets of the wild type *N. perurans* isolates displayed in Fig. 3 can be found in Table S4.

## Discussion

Therapeutic treatments in contemporary Atlantic salmon mariculture, including freshwater bathing and hydrogen peroxide bathing, persist as the primary commercially viable methods of AGD control^7,32^. While development of alternative AGD control methods has largely been hindered by the limited progress made towards identifying *N. perurans* virulence determinants, advances in omics-based approaches have provided valuable tools by which therapeutic targets and vaccine candidates can be identified. Here, leveraging comparative transcriptomic analysis of confirmed virulent and avirulent *N. perurans* isolates, four categories of putative virulence determinants, encompassing genes encoding for cytoskeleton reorganisation, response to oxidative stress, protease activity, and lipid metabolism were identified.

Dynamic reorganisation of the cytoskeleton, typically by actin-myosin mediated processes, is necessary for cell motility and phagocytosis in amoeboid eukaryotes and, by extension, host colonisation by parasitic amoebae^33,34^. Here, cytoskeleton, cell motility, phagocytosis, and pseudopodium associated gene sets, of which actin is an essential component, were amongst the most highly enriched in the wild type isolates. Accordingly, an actin homologue (*Physarum polycephalum*), β-actin homologue (*Paramoeba pemaquidensis*), and two myosin-1 homologues (*Acanthamoeba castellanii*) were highly expressed in both clonal and wild type isolates, although significantly upregulated in the wild type amoebae. Considered a pathogenicity factor in other parasitic amoebae^35,36^, knockdown of actin gene expression of *N. pemaquidensis* has been shown to result in trophozoite transformation into an immobile cyst-like state^37^, suggesting actin-mediated processes are essential in supporting functions necessary for virulence in *N. perurans*.

To support the dynamic reorganisation of the cytoskeleton, actin-binding proteins are required to regulate actin assembly^34^. Three gene sets associated with actin assembly regulation, including actin-filament binding, actin crosslink formation, and actin binding were highly enriched in the wild type isolates. Within these gene sets, homologues of severin and gelsolin/villin family proteins were upregulated. Severin and villin appear to play a role in cytoskeleton dynamics^38,39^ and upregulated expression of these proteins has previously been observed in highly virulent cultures of *N. fowleri*^40,41^. Upregulated gene expression of profilin and formin homologues were also observed in the wild type amoebae. Profilin and formin are necessary for formation and elongation of actin filaments^42^ and have been shown to play an essential role in pseudopodia formation and phagocytosis in *E. histolytica*^43^, which correlate with its in vivo capacity for virulence^44^. While the association between *N. perurans* phagocytic capacity and AGD pathogenesis has not been determined^31^, a defining characteristic of clonal *N. perurans* avirulence is the incapacity for host attachment and associated initiation of AGD in Atlantic salmon^29^. Ultrastructural evidence suggests that *N. perurans* pseudopodia may extend into the gill epithelium^45,46^, potentially facilitating a form of host attachment, and implicating presumably actin-myosin mediated processes in AGD pathogenesis.

Eukaryotic elongation factors are highly conserved house-keeping polypeptides involved in protein synthesis and a variety of other cellular processes^47^, identified as protective vaccine candidates against several protozoan parasites^48,49^. Two transcripts putatively encoding for elongation factor 1 alpha (eEFA1; *Paramoeba branchiphila*) and elongation factor 2 (eEF2; *Colpoda inflata*) were exclusively expressed in the wild type isolate, along with highly upregulated expression of a bacterial elongation factor thermo unstable (EF-Tu; *Novosphingobium aerophilum*) homologue. Protein homologues of eEFA1 and potentially EF-Tu have previously been identified in *N. perurans* isolates^27^. Functionally, *Neoparamoeba* spp. eEFA1 appears to play a role in cell cytoskeleton dynamics, with knockdown using recombinantly produced *N. pemaquidensis* eEFA1 demonstrated to influence cell shape, and to reduce cell motility and pseudopodia radiation^37^. Similar eEFA1 function has been observed in *E. histolytica*, with co-localisation of eEFA1 and actin in pseudopodia and at sites of adhesion suggested to promote phagocytosis and play a role in pathogenicity^50^. Considering the promising protective effect provided by eEFA1 vaccine candidates against other parasitic protozoans^48,49^, the upregulated expression of putative elongation factors in wild type *N. perurans* isolates warrants further investigation of their antigenic properties and potential role in pathogenicity.

Antioxidant enzymes constitute an established defence mechanism that protects parasites from endogenously produced and exogenous sources of reactive oxygen species^51^. Colonisation of *N. perurans* stimulates gill infiltration with inflammatory immune cells^5,46^, wherein a key process of host phagocyte antimicrobial activity is the production of reactive oxygen species^52^. Here, four transcripts with homology to the superoxide dismutases and two transcripts with homology to peroxiredoxins were highly expressed in both the wild type and clonal isolates, with significant upregulated expression observed in the wild type isolates. While the expression of superoxide dismutases has previously been reported in transcriptomic^30^ and proteomic analyses^27,28^ of xenic cultures of virulent *N. perurans*, the current study is the first to report gene expression of *N. perurans* peroxiredoxin homologues. Expression of peroxiredoxins and superoxide dismutases, primarily through enzyme mediated protection against host immune effector cells, have been attributed to pathogenicity in other parasitic amoebae species, including *E. histolytica*^53,54^, *N. fowleri*^55^ and *A. castellanii*^56^. Trophozoite survival during gill colonisation under host-mediated inflammatory conditions and with exposure to the gill-localised oxidative stress associated with AGD progression^57^ is indicative of the significance of endogenous antioxidant production in *N. perurans* pathogenicity.

Extracellular proteases are commonly described determinants of virulence in parasitic amoeba species that enable host invasion by facilitating host cell destruction and immune cell degradation^58–60^. As reported in these species, a contact-independent cell cytopathic effect has also been observed in vitro in *N. perurans*^29^. Previous proteomic analysis has implicated an S8 and S53 domain containing protein in this extracellular cytopathic effect, although attributed to virulence-attenuated *N. perurans* cultures, while highlighting the absence of its expression in virulent *N. perurans* cultures^28^. In contrast, in the current study, gene expression of the *N. perurans* peptidase S8, and S53 domain-containing protein was significantly upregulated in the wild type isolates relative to clonal *N. perurans* cultures. Here, cell lysis and subsequent RNA extraction from wild type isolates was performed directly after gill isolation, as opposed to gill isolation and subsequent 70-day xenic MYA culture of *N. perurans* prior to processing as previously described^28^. Although based on the implicit assumption that differential mRNA expression reflects protein level differences, wild type gene expression of proteases putatively associated with *N. perurans* cell cytotoxicity is indicative of external stimuli mediating production of extracellular product. Host stimulated secretion of cytopathic proteases has been observed in *A. castellanii* following exposure to host cell surface mannose residues^59^. Nonetheless, further in vitro validation of the potential need for external stimuli in production of wild type *N. perurans* extracellular product is required.

Cathepsins are a family of potent lysosomal proteases, of which some members retain activity outside of the endo/lysosomal system, that potentially support host invasion, and nutrient uptake in several parasitic amoeba species^60,61^. Through association with the putative role of these proteases in parasitic amoeba pathogenicity, expression of cathepsin B homologues in *N. perurans* has been suggested to play a role in AGD pathogenesis^30^. Here, gene expression of a cathepsin B domain containing protein with homology to *N. fowleri* cathepsin B, and two additional aspartic and cysteine cathepsin homologues, were upregulated in the wild type isolates. *Naegleria fowleri* cathepsin B, while localised in food cups and pseudopodia, is readily secreted and has been implicated in contact-independent pathology^60,62^. Notably, in previous research, cathepsin family protein expression was absent from the extracellular product of both clonal and wild type *N. perurans* cultures^28^, suggesting that these proteases do not play a role in excretory-secretory host cell lysis as observed in other parasitic amoeba. However, given the relevance of cathepsins as therapeutic or vaccine targets against parasites^63,64^, the upregulated expression of genes encoding for putative cathepsins in wild type *N. perurans* warrants further investigation of the potential role of these proteases in AGD pathogenesis.

Fatty acid oxidation encompasses a subset of catabolic processes in fatty acid metabolism that generate energy for cellular function^65^. Here, lipid oxidation, including gene sets associated with fatty acid beta oxidation and acyl-CoA oxidase activity, was highly enriched in the wild type isolates. While no studies to date have reported information regarding *N. perurans* fatty acid metabolism, the closely related *N. pemaquidensis* and endosymbiont, *Perkinsela sp*., appear to encode a complete set of genes required for fatty acid β-oxidation involving mitochondria and a glycosome/peroxisome-like organelle^66^. Previous research has shown in vitro that *N. gruberi* has an energy substrate preference for fatty acid oxidation^67^. Predicted to have the same preference, upregulation of genes associated with fatty acid oxidation has previously been observed in *N. fowleri* following mouse passage^40^, and inhibition of fatty acid oxidation using approved therapeutics has been demonstrated to impede trophozoite growth *in vitro*^68^. Currently, it is not known if fatty acid oxidation is necessary for *N. perurans* viability. However, the upregulated expression of genes putatively involved in *N. perurans* fatty acid oxidation highlights fatty acids as a potentially important energy substrate during gill colonisation and AGD pathogenesis.

Comparative transcriptomic analysis of virulent, gill-associated wild type *N.* *perurans* and confirmed avirulent, clonal *N.* *perurans* cultures revealed distinct gene expression profiles between the two phenotypes. Through identification of upregulated gene expression in wild type *N. perurans* and subsequent GSEA, genes putatively encoding for *N. perurans* virulence determinants were categorised under cytoskeleton reorganisation, response to oxidative stress, protease activity, and lipid metabolism. Given the paucity of knowledge concerning *N. perurans* biology and AGD pathogenesis, many of the putative virulence determinants presented here were inferred from other parasitic amoeba species. While the identification of potential therapeutic and vaccine candidates provides a foundation for the development of hypotheses for future AGD research, the necessity of further investigation of the mechanisms by which *N. perurans* induce AGD is emphasised.

## Methodology

### *Neoparamoeba perurans* culture and sample collection

Clonal and wild type *N. perurans* isolates were obtained from the University of Tasmania (Launceston, Tasmania, Australia). Clonal (clone 4) *N. perurans* were cultured on MYA plates as described previously^69^ for approximately 1,095 days prior to use in the current study and have been demonstrated to be avirulent^29^. Wild type *N. perurans* were isolated directly from the gills of Atlantic salmon derived from commercial stock, held at the University of Tasmania Aquaculture Centre (Launceston, Tasmania) using previously published methods^70^. Three separate wild type isolations were obtained from AGD mortality events over a period of one week. Each isolation was made up of 6 × 10^5^ to 9 × 10^5^ amoebae. Isolated wild type trophozoites were not subjected to MYA plate culture. Following gill isolation, wild type *N. perurans* were concentrated by centrifugation (550 g, 6 min at 4 °C) and immediately lysed with a lysis buffer (4 M Urea, 0.5% SDS, 50 mM Tris, 10 mM EDTA) and retained for further processing.

Clonal *N. perurans* isolations were pooled from multiple culture plates, with each isolation consisting of 5 × 10^4^ to 3 × 10^5^ amoebae. To harvest clonal *N. perurans* from MYA agar plates, amoebae were dislodged from the agar with gentle agitation using a plate spreader. The resulting supernatant was collected in a 50 mL microcentrifuge tube and vortexed for 3 s. The supernatant was then placed on a magnetic stirrer at half speed for 3 min to dislodge amoebae trapped in viscous culture medium. The supernatant was then centrifuged at 550 g for 5 min at room temperature to concentrate. After the supernatant was discarded, sterile seawater was added, and the pellet resuspended by gentle agitation. From this, 2 mL was transferred into individual wells of 6-well cell culture plates and amoebae were allowed to attach for 30 min. The supernatant was then discarded, attached amoebae were washed with sterile sea water, and double concentration lysis buffer immediately added to lyse cells for further processing.

### Total nucleic acid and RNA extraction

Total RNA was extracted from *N. perurans* trophozoites using lysis buffer and purified using TRIzol Reagent (Thermo Fisher Scientific, Australia), with an on-column DNase treatment step (Baseline-ZERO DNase, Lucigen, USA), according to the manufacturer’s protocol. Total RNA yields were determined spectrophotometrically using Qubit broad range RNA assays (ThermoFisher, Australia), and RNA integrity was estimated using gel electrophoresis on 1% agarose gel. For sequencing, from pooled wild type and clonal *N. perurans* RNA samples, 3 × 1.84 µg aliquots of wild type isolate RNA, and 3 × 1.70 µg aliquots of clonal isolate RNA were prepared and stored as ethanol precipitates (2.5 M ammonium acetate, 100% ethanol).

### RNA sequencing and De novo transcriptome assembly

Purified RNA from clonal (n = 3) and wild type (n = 3) *N. perurans* isolates was delivered to Macrogen Inc., for library preparation for RNA sequencing (RNAseq). The sequencing workflow consisted of mRNA library preparation using reagents provided in the Illumina TruSeq Stranded mRNA LT sample prep kit following the protocol, TruSeq Stranded mRNA sample preparation guide, part #15,031,047 Rev. E. Sequencing was conducted using an Illumina Hiseq system as 101 base paired-end reads, with an input quantity of 1 µg total RNA per sample.

Prior to transcriptome assembly, the quality of raw RNAseq reads was assessed using FastQC (www.bioinformatics.bbsrc.ac.uk/projects/fastqc). Random sequencing errors were corrected with rCorrector^71^. In read pairs where at least one read was deemed unfixable, the “FilterUncorrectabledPEfastq.py” python script was used to discard these unfixable read pairs and to remove the “cor” header tags for downstream analysis (https://github.com/harvardinformatics/TranscriptomeAssemblyTools). Residual adapter sequences and low-quality bases (phred < 5) were trimmed using the Trim Galore! wrapper application^72^, with read pairs equal to or longer than 36 bp retained post-filtering. As wild type *N. perurans* were harvested from the gills of AGD-affected Atlantic salmon prior to RNA extraction, STAR ^73^ was used to map wild type and, to remain consistent, clonal *N. perurans* RNAseq reads to the Atlantic salmon genome assembly (ICSASG_v2; Genbank accession GCF_000233375.4) to remove host sequences. Residual rRNA was removed from RNAseq reads by mapping to the SILVA rRNA database^74^ using Bowtie2^75^. Processed RNAseq reads from three wild type and three clonal samples were then assembled with Trinity^76^ using default parameters.

### Assembly quality and transcript abundance estimation

Read support for the assembled transcriptome was quantified by mapping paired end RNAseq reads back to the assembled transcriptome using Bowtie2. For quantitative assessment of de novo transcriptome assembly quality and completeness, the presence of Benchmarking Universal Single-Copy Orthologs (BUSCO) was analysed using the conserved eukaryotic proteins (eukaryota_odb10, created 2020–09-10) database. To reduce the proportion of reported duplicates, the transcriptome assembly was filtered for isoforms prior to BUSCO analysis. After assessment of transcriptome quality, alignment-free transcript abundance was quantified by mapping filtered reads in Fastq format to the assembled transcriptome using Salmon^77^. A gene expression matrix was constructed using the “abundance_estimates_to_matrix.pl” python script included in the Trinity package.

### Transcriptome annotation

Transcriptome assembly annotation was performed using Trinotate^78^. In brief, putative coding regions were extracted from the transcriptome assembly using TransDecoder (http://transdecoder.github.io) with default parameters and annotated by mapping transcripts and these putative coding regions separately using Blast leveraging Swiss-Prot and pfam databases. The NCBI NR database was included as a custom Blastx search using diamond with the “–very-sensitive” command line option^79^. All Blast searches were conducted with an expect value (e-value) cut-off of < 1 × 10^–5^, with the top blast hit used for annotation. Gene Ontology (GO) terms were assigned from best-matching Swiss-Prot annotations of homologous proteins, pfam2go, and eggnog database mapping. A custom gene matrix transposed (gmt) file was produced by extracting GO assignments from the Trinotate output file using the “extract_GO_assignments_from_Trinotate_xls.pl” script from the Trinity package. The resulting file was formatted for GSEA using a custom perl script.

### Bioinformatic analyses

Bioinformatic analyses were performed in R v4.02^80^ and the GSEA desktop application v4.1.0^81^ at the gene level using genes predicted by Trinity. The RNAseq dataset was evaluated for significant outliers using both principal component analysis (PCA) and hierarchical clustering. Differential gene expression analysis was conducted using DESeq2^82^, with gene-level abundance count data normalisation using the variance stabilising transformation (VST) function performed for downstream exploratory analysis, and gene set enrichment analysis (GSEA). The Wald test was used to generate *p* values and Log2 fold changes comparing the wild type to clonal *N. perurans* isolates. Genes with Benjamini–Hochberg false discovery rate (FDR) adjusted *P* values less than 0.05 and a log2 fold change (Log2FC) equal to or greater than one were considered differentially expressed. Gene set enrichment analysis^81^ was used to identify gene sets with statistically significant differences in expression between the wild type and clonal *N. perurans* isolates. All transcripts putatively assigned to genes by trinity, presented as a gene count matrix normalised using variance stabilising transformation (VST), were subjected to GSEA using a custom GMT file (produced as described in Sect. "Transcriptome annotation") as the reference gene sets file. Default parameters were used for GSEA, with gene sets smaller than 15 and larger than 500 excluded and significance tested using gene set permutation (2500 permutations). Results from GSEA were visualised using the Enrichment Map plugin for Cytoscape^83^ with default parameters.

## Supplementary Information

Supplementary Information 1.

Supplementary Information 2.

Supplementary Information 3.

Supplementary Information 4.

## Data Availability

Data supporting this study are included within the article, the supplementary material, and the National Center for Biotechnology Information (NCBI) Sequence Read Archive (BioProject: PRJNA800826).
