# Supplementary material for: Comparative transcriptome profiling of virulent and avirulent isolates of Neoparamoeba perurans
Source: Sci Rep. 2022 Apr 7;12:5860. doi: 10.1038/s41598-022-09806-5 (PMC8989968; doi:10.1038/s41598-022-09806-5)
Supplement: Supplementary file 1 — Supplementary Information 1. [file 41598_2022_9806_MOESM1_ESM.pdf]

Comparative transcriptome profiling of virulent and avirulent isolates of *Neoparamoeba perurans*

Aaron J. Smith*, Philip B.B. Crosbie, Barbara F. Nowak, Andrew R. Bridle

Institute for Marine and Antarctic Studies, University of Tasmania, Launceston, Tasmania, Australia

*Corresponding author

Email: aj.smith@utas.edu.au

Supplementary table S1A. Neoparamoeba perurans transcriptome assembly statistics

**Trinity *de novo* assembly**	
Trinity genes	66,361
Trinity transcripts	86,517
Total assembled bases	68,135,905
Percent GC	47.07
Contig N50	1,155
Contig E90 # transcripts	8,038
Contig E90 N50	1,307
BUSCO % completeness	84.7

Supplementary table S1B. Raw data from RNAseq samples pre-filtering

Accession	Sample	Total bases	Read count	GC (%)	Q20 (%)	Q30 (%)
SRR17772868	Clone4_-1_1	3,529,311,276	34,943,676	51	98.32	91.25
SRR17772868	Clone4_-1_2	3,529,311,276	34,943,676	51	97.62	92.50
SRR17772867	Clone4_-2_1	3,324,748,199	32,918,299	51	98.31	91.34
SRR17772867	Clone4_-2_2	3,324,748,199	32,918,299	51	97.73	92.73
SRR17772866	Clone4_-3_1	2,844,160,707	28,160,007	50	98.34	91.47
SRR17772866	Clone4_-3_2	2,844,160,707	28,160,007	50	97.54	92.53
SRR17772865	Wild_type_-1_1	3,249,881,242	32,177,042	52	98.44	91.74
SRR17772865	Wild_type_-1_2	3,249,881,242	32,177,042	51	97.85	92.97
SRR17772864	Wild_type_-2_1	3,039,286,647	30,091,947	51	98.36	91.61
SRR17772864	Wild_type_-2_2	3,039,286,647	30,091,947	51	97.80	92.88
SRR17772863	Wild_type_-3_1	3,327,365,008	32,944,208	52	98.43	91.75
SRR17772863	Wild_type_-3_2	3,327,365,008	32,944,208	52	97.74	92.96

*_1 or _2 (e.g., Clone4_-1_1) denotes paired end read direction; Clone4 = avirulent, Wild type = virulent

Supplementary table S1C. Filtered RNAseq (processed) data used in transcriptome assembly

Sample	Total bases	Read count	% reads survived filter	GC (%)	Q20 (%)	Q30 (%)
Clone4_-1_1	3,387,385,926	34,555,846	95.98	51	99.9996	95.27
Clone4_-1_2	3,390,876,706	34,555,846	96.08	51	99.9876	95.50
Clone4_-2_1	3,190,351,919	32,563,010	95.96	51	99.9993	95.34
Clone4_-2_2	3,194,901,215	32,563,010	96.09	51	99.9894	95.66
Clone4_-3_1	2,728,859,007	27,812,188	95.95	50	99.9996	95.41
Clone4_-3_2	2,731,259,875	27,812,188	96.03	50	99.9884	95.58
Wild_type_-1_1	3,147,618,307	31,944,618	96.85	52	99.9998	95.46
Wild_type_-1_2	3,152,098,791	31,944,618	96.99	52	99.9908	95.67
Wild_type_-2_1	2,916,491,423	29,783,253	95.96	51	99.9996	95.45
Wild_type_-2_2	2,920,145,637	29,783,253	96.08	51	99.9911	95.71
Wild_type_-3_1	3,216,134,710	32,652,595	96.66	52	99.9997	95.43
Wild_type_-3_2	3,221,713,707	32,652,595	96.82	52	99.9901	95.76

*_1 or _2 (e.g., Clone4_-1_1) denotes paired end read direction; Clone4 = avirulent, Wild type = virulent
